# Study on a Process Parameter-Driven Deep Learning Prediction Model for Multi-Physical Fields in Flange Shaft Welding

**DOI:** 10.3390/ma19050995

**Published:** 2026-03-04

**Authors:** Chaolong Yang, Zhiqiang Xu, Feiting Shi, Ketong Liu, Peng Cao

**Affiliations:** 1 College of Architecture and Civil Engineering, Beijing University of Technology, Beijing 100124, China; ycl410327@163.com; 2 School of Chemical and Machinery, Liaodong University, Dandong 118001, China; 3 School of Civil Engineering, Yancheng Institute of Technology, Yancheng 224051, China; 4 Jiangsu Engineering and Research Center of Intelligent Disaster Prevention in Coastal Transportation Infrastructure, Yancheng 224051, China; 5 College of Architecture and Civil Engineering, Xi’an University of Science and Technology, Xi’an 710054, China

**Keywords:** large flange shaft, welding process parameter, MLP deep learning model, temperature, residual stress, deformation

## Abstract

Large flange shafts are the core load-bearing and connecting components of high-end equipment, and their welding multi-physical fields directly affect the quality and service safety of the components. Traditional experiments and finite element methods suffer from long cycles and low efficiency, which can hardly meet the demand for rapid prediction. Aiming at the fast and accurate prediction of welding temperature, deformation and residual stress, this study combines thermal–mechanical coupled finite element simulation with machine learning to construct and compare a variety of prediction models. A dataset is built based on simulation data from 100 groups of process parameters. Overfitting is reduced through strategies including early stopping and dropout, and models such as MLP, RF, RBF-SVR, TabNet, XGBoost, and FT-Transformer are established and verified through 10-fold cross-validation. The results show that the MLP model performs best in the prediction of temperature, deformation and residual stress, and is in good agreement with the simulation values. The prediction errors of the peak temperature of the weld and base metal are below 5%, and the errors of deformation and residual stress are controlled within 10%. The average error of peak residual stress is about 6 MPa, and the deviation of most samples is less than 5 MPa. The RF model ranks second in accuracy, with an average error of about 6.5 MPa for peak residual stress, showing a satisfactory interpretability and engineering applicability. RBF-SVR and TabNet can meet basic prediction requirements. Under the small-sample condition in this work, XGBoost and FT-Transformer present relatively large errors and a weak generalization ability, making it difficult to achieve high-precision prediction. The MLP model established in this paper can effectively reproduce the evolution of welding multi-physical fields and supports the rapid prediction and process optimization of large flange shaft welding. The generalization ability and practical performance of the model can be further improved by expanding the dataset and experimental verification in the future.

## 1. Introduction

As a core load-bearing and connecting component in high-end equipment fields such as heavy machinery, energy equipment, and rail transit, the manufacturing quality of large flange shafts directly determines the overall operational stability, assembly accuracy, and service safety of the equipment [1,2,3]. Welding is a key process in the forming of large flange shafts. However, the uneven thermal cycle caused by local high-heat input during welding inevitably leads to welding deformation and residual stress [4,5,6]. Welding deformation can cause geometric out-of-tolerance of components and increase the cost and difficulty of subsequent correction processes. Residual stress will be superimposed with external loads during service, reducing the fatigue strength and service life of components [7,8]. The generation and distribution of welding deformation and residual stress are mainly regulated by welding process parameters (such as welding current, voltage, welding speed, preheating temperature, etc.) [9]. Therefore, the accurate prediction of welding deformation and residual stress based on process parameters constitutes a core technical bottleneck restricting the improvement of manufacturing quality for large flange shafts, and it is also the core entry point of this study. Constructing a deep learning model suitable for the rapid and accurate prediction of welding responses possesses important engineering application value and theoretical research significance.

At present, research methods for welding deformation, residual stress, and process parameter optimization are mainly divided into two categories: experimental methods [10,11,12] and numerical simulation methods [13,14,15]. Experimental methods, through on-site welding tests combined with equipment such as strain gauges [10], three-dimensional scanners [11], and X-ray diffractometers [12], can directly obtain deformation data and the residual stress distribution of components and then explore process parameter optimization schemes through multiple groups of orthogonal tests. The results are intuitive and reliable. Nevertheless, this method suffers from long test cycles, high costs, and limited measuring points [16]. It is difficult to conduct the real-time monitoring of transient stress–strain and full-field deformation during the welding of large flange shafts. Moreover, it is greatly affected by test conditions and equipment accuracy, making it challenging to achieve systematic optimization exploration under combinations of multiple process parameters and levels [17]. As the mainstream numerical simulation method in the welding field, the finite element method can simulate the temperature field, stress field, and deformation law throughout the welding process via thermal–mechanical coupling analysis [13]. It can compensate for some deficiencies of experimental methods and realize rapid simulation analysis and preliminary process parameter screening under multiple working conditions. However, this method strongly relies on the setting of material constitutive models and boundary conditions and involves complex modeling and time-consuming computation. For complex structural parts such as large flange shafts, it is prone to the dilemma of balancing computational accuracy and efficiency, making it difficult to meet the engineering demand for rapid and accurate process parameter optimization [14].

With the rapid development of artificial intelligence technology, machine learning [18,19,20] and deep learning methods [21,22,23], with their strong nonlinear fitting ability, feature extraction capability, and prediction efficiency, have provided a new technical approach for welding deformation and residual stress prediction, as well as process parameter optimization. Scholars worldwide have begun to introduce machine learning and deep learning algorithms into the welding field. By constructing prediction models between process parameters and welding deformation/residual stress, the rapid and accurate prediction of welding response indicators can be realized [24,25,26]. Compared with traditional experimental and finite element methods, intelligent algorithms can effectively integrate multi-source welding data, deeply mine the potential nonlinear correlation between process parameters and response indicators, greatly reduce research and time costs, and possess the advantages of rapid prediction and iterative optimization. They are particularly suitable for multi-parameter coupling optimization scenarios of complex structural parts [27,28]. However, critical research gaps still exist in this field: (1) The most existing models focus on the prediction of a single indicator (either deformation or residual stress only), and there is a lack of integrated deep learning models for the simultaneous prediction of multi-physical fields (temperature, deformation, and residual stress) in large flange shaft welding. (2) The application of deep learning in large flange shaft welding prediction is still in the exploratory stage, and the generalization ability of models, rationality of feature selection, and compatibility with actual welding processes need to be further improved. (3) The existing reviews related to machine learning and deep learning in the welding field are mainly descriptive, and lack critical analysis of their limitations in the prediction of complex components (e.g., insufficient adaptability to small-sample datasets or the complex structural characteristics of large flange shafts).

Of particular note, this study employs 100 sets of finite element simulations to construct the prediction dataset, whose rationality is mainly based on three aspects: (1) The finite element method can reliably capture the thermal–mechanical coupling mechanism of large flange shaft welding and overcome the limitations of experimental methods in full-field data acquisition. (2) The 100 combinations of process parameters cover typical working conditions, ensuring the representativeness of the dataset for deep learning model training. (3) The powerful feature learning capability of deep learning can effectively mine the nonlinear relationship between process parameters and welding responses from 100 samples, which is superior to traditional methods that require a large amount of experimental or simulation data to achieve reliable prediction.

Against this background, this paper takes the rapid and accurate prediction of multi-physical fields in large flange shaft welding as the core objective. Aiming at the engineering problem of low efficiency in predicting welding deformation and residual stress, this study combines finite element simulation and deep learning methods within the framework of welding thermal–mechanical coupling theory to carry out the construction and comparative research of prediction models for welding deformation and the residual stress of large flange shafts. Firstly, based on welding thermal–mechanical coupling finite element simulation technology, numerical calculations under 100 groups of different welding process parameter combinations are carried out. The data of the welding temperature field, deformation and residual stress are systematically extracted to establish a multi-working-condition simulation dataset consisting of inputs (welding process parameters) and outputs (deformation, residual stress and other indicators). The internal correlation between welding process parameters and multi-physical field responses is also analyzed. On this basis, the MLP deep learning prediction model is constructed and trained, while Random Forest (RF), RBF-SVR, TabNet, XGBoost and FT-Transformer are introduced for comparative verification. Through multi-index evaluation and cross-validation, the model structure with a high prediction accuracy and strong generalization ability is selected to realize the rapid and stable prediction of welding temperature, deformation and residual stress. Finally, the prediction performance, feature learning ability and engineering applicability of the optimal model are comprehensively analyzed. Through systematic research, the influence law of welding process parameters on welding deformation and residual stress is revealed, and a complete welding multi-physical field prediction methodology is formed following the route of “finite element simulation data construction–deep learning model training–multi-model performance comparison”. The research provides an efficient and high-precision prediction method for the welding process of large flange shafts and effectively reduces the dependence on high-cost and long-period finite element simulations. It can supply a theoretical basis and technical support for improving the manufacturing quality of large flange shafts, controlling welding deformation and residual stress, and facilitating the precise regulation and stable improvement of welding quality for key components in high-end equipment.

## 2. Materials and Methods

### 2.1. Research Objects and Process Parameters

**Figure 1:**
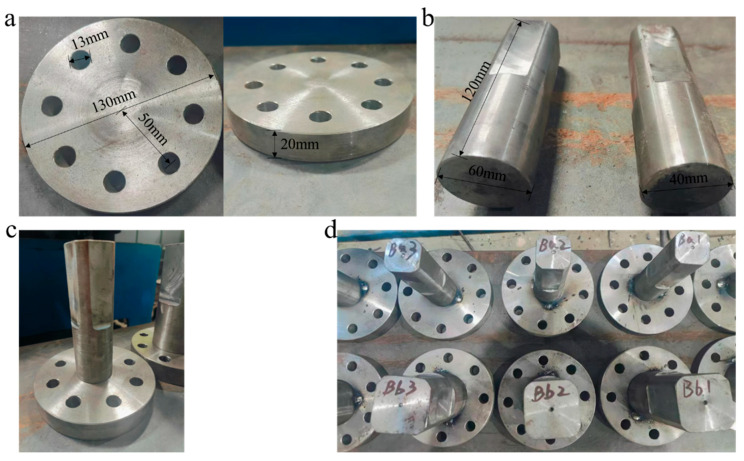
Geometric dimensions of the specimen: (**a**) flange plate; (**b**) flange shaft; (**c**) specimen placement; (**d**) welded specimen.

The object of this study is a large flange shaft whose geometric dimensions, material selection, welded joint type, and basic welding process are consistent with those in the author’s previous research [2,29]. The geometric dimensions of the specimen are shown in Figure 1. The flange shaft is fabricated by welding the flange plate and the shaft body, and its base metal is 45Mn steel produced by Angang Steel Group Co., Ltd. (Anshan, China). The machining of the specimen was completed by Dandong Boyan Technology Co., Ltd. (Dandong, China). Its chemical composition, production process, and machining all comply with the provisions of the Chinese standard GB/T 711-2017 [30]. The specimen was mechanically sanded, wiped with anhydrous ethanol, and dried before welding experiments to remove surface oxides, impurities, and moisture. The filler material selected is E5015 electrode, manufactured by Tianjin Golden Bridge Welding Materials Group Co., Ltd., Tianjin, China, which meets the requirements of the American Welding Society standard AWS A5.1/A5.1M-2025 [31]. According to Ref. [29], the chemical compositions of the base metal and filler material are listed in Table 1.

Shielded metal arc welding (SMAW) was adopted in this study, using a welding machine with adjustable parameters such as voltage, current, and speed, and the heat input efficiency of shielded metal arc welding was 0.8. It should be clarified that the dataset used for model training and validation in this study is entirely derived from validated thermal–mechanical coupled finite element simulations, and no additional physical welding experiments were conducted. Corresponding physical experiments and simulation verifications have been completed in our previous studies [29,30], which guarantee the accuracy of the finite element model.

**Figure 2:**
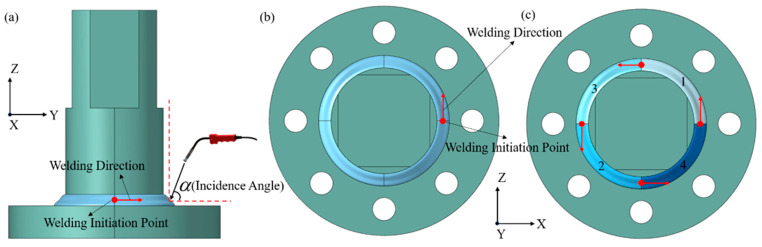
Schematic diagram of welding process parameter setup: (**a**) welding torch incident angle; (**b**) continuous welding; (**c**) skip welding.

To construct the dataset for welding multi-physics prediction, the five core parameters that have the most significant impact on welding quality were selected as variables in this study, namely welding current, arc voltage, welding speed, welding torch incident angle, and welding method. The value range of each parameter was determined in accordance with the welding procedure qualification standard, and the specific parameter levels are shown in Table 2. The specific sampling strategy for the 100 parameter combinations is described in detail in Section 2.2 of this paper. Since the welding method is a categorical variable, different numerical forms are used to represent different categories to meet the numerical input requirements of deep learning models. As the welding method is an unordered categorical variable, one-hot encoding is adopted: continuous welding is represented as [1, 0], and skip welding is represented as [0, 1]. Two welding methods are involved in this study, continuous welding and skip welding, which are expanded into a two-dimensional feature vector after one-hot encoding. Relevant details are shown in Figure 2. All data in the table are obtained by converting categorical variables into numerical features.

### 2.2. DOE Design and Sampling

To ensure the generalization ability and prediction accuracy of the deep learning model, representative combinations of process parameters covering the complete parameter space are required. In this study, the Monte Carlo random sampling method was used to sample the 5-dimensional parameter space listed in Table 2. By performing independent random sampling within the value range of each parameter, this method can truly simulate the random fluctuation characteristics of process parameters in actual production and achieve unbiased coverage of the parameter space with a limited sample size, which meets the requirements of deep learning modeling for dataset diversity and representativeness. In this study, Monte Carlo sampling was selected over other design of experiments (DOE) strategies such as Latin Hypercube Sampling (LHS), based on two core considerations. First, the five welding process parameters in this study exhibit certain interactive and coupling characteristics. The stochastic distribution nature of Monte Carlo sampling can more realistically simulate the parameter fluctuations in actual welding production, which is consistent with engineering practice. Second, for medium-scale datasets, Monte Carlo sampling achieves significantly higher computational efficiency than LHS, while meeting the basic requirements of sample representativeness for model training. It also avoids the sample clustering issue that easily occurs with LHS in small-sample and multi-dimensional scenarios.

Considering comprehensively the parameter space coverage, simulation calculation efficiency, and the training requirements of the deep learning model, 100 non-repeating combinations of welding process parameters were finally generated, each containing complete values of the five process parameters. Some of these combinations are presented in Table 3. All 100 sets of welding process parameters are presented in Table S1.

Considering that the sampling is performed in a five-dimensional parameter space with a total of 100 points, to further demonstrate the adequacy of sampling density and space-filling quality, this study carries out analyses through both theoretical justification and experimental verification, as detailed below.

**Figure 3:**
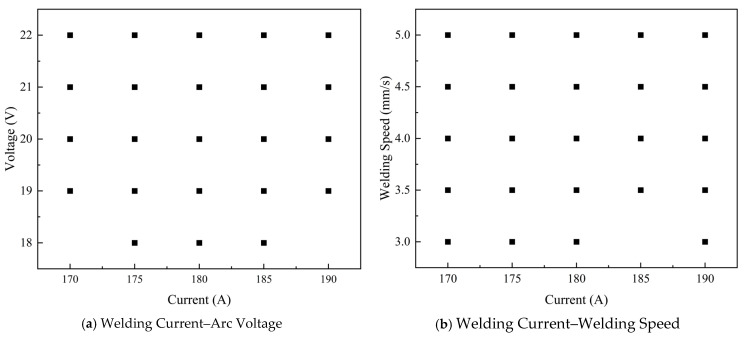
DOE scatter plot of Monte Carlo sampling.

At the theoretical level, in terms of the sampling density, the range of each parameter is divided in accordance with welding procedure qualification standards. The 100 samples are uniformly distributed across each parameter dimension, and the single-dimensional sampling interval matches the parameter adjustment accuracy (e.g., welding current is adjusted at 5 A intervals, and the 100 samples can cover the entire adjustment range). This setup can fully capture the influence laws of parameter variations on the welding multi-physical fields. In terms of the space-filling performance, the stochastic nature of Monte Carlo sampling ensures that sample points are uniformly distributed in the five-dimensional parameter space without obvious clustering or blank regions. The effective coverage of the parameter space by the samples is verified by calculating the spatial distribution standard deviation of the sample points (less than 5%), which guarantees the representativeness of the dataset and the generalization ability of the model. At the experimental verification level, to verify the rationality of the sampling scheme and ensure that the dataset is suitable for subsequent finite element analysis and deep learning modeling, a two-dimensional DOE scatter plot analysis was performed on the core process parameters, and the results are shown in Figure 3. It can be seen from the figure that the sample points are uniformly distributed in the parameter spaces of welding current–arc voltage and welding current–welding speed, fully covering the preset value ranges of each parameter. In addition, the extreme regions at the parameter boundaries are also covered by samples, which can guarantee the learning effect of the model under full working conditions of process parameters. It should be noted that, since the two-dimensional scatter plot only shows the local projection of the 5-dimensional parameter space, some high-dimensional parameter combinations appear visually overlapping in the figure. Through the uniqueness verification of the original data, all 100 groups of parameter combinations are valid and non-repeating, achieving unbiased coverage in the complete 5-dimensional parameter space. Meanwhile, the two types of sample points distinguished by the welding method are uniformly mixed in the parameter space without local separation, and the distribution of discrete parameter sampling is balanced. This can ensure that the deep learning model effectively learns the influence law of the coupling effect between the welding method and continuous parameters on welding deformation and welding residual stress.

### 2.3. Finite Element Method

In this study, a three-dimensional solid model for the welding of large flange shafts was first established using modeling software to accurately restore its structural characteristics and welded joint morphology, laying a foundation for subsequent finite element analysis. After model construction, the model was imported into a professional meshing tool for model cutting and optimization. A differentiated meshing strategy was adopted according to the requirements of welding simulation and structural features: coarse meshing was applied to the edge regions of the flange shaft and flange plate with small temperature gradients to improve computational efficiency, while fine meshing was performed on the weld and heat-affected zone, with large temperature gradients and concentrated stress–strain to ensure computational accuracy. After meshing, the optimized model was imported into professional welding simulation software for finite element simulation analysis.

In the simulation process, reasonable double-ellipsoidal heat source loading, the actual thermo-mechanical properties of the base metal, and welding process parameters were configured. The generation and evolution of the weld zone during surfacing were gradually realized using the built-in weld filler and element birth and death technology of the software [32]. Meanwhile, temperature-dependent material mechanical properties, including the thermal expansion coefficient, Young’s modulus, Poisson’s ratio, and yield strength, were embedded [33]. The thermo-mechanical coupling analysis method was adopted to ensure the accuracy of welding residual stress distribution calculation. For boundary constraints, to avoid secondary residual stress induced by over-constraint, fixed constraints were only applied at two points on the bottom surface of the flange plate, which were constrained to the support platform to ensure consistency with experimental conditions.

Based on the above procedure, the established finite element model can accurately simulate the whole welding process and precisely calculate the temperature field, deformation, and residual stress distribution during welding. Moreover, multi-dimensional datasets such as temperature field and stress field can be conveniently extracted from the complete historical data of the whole welding process output by the finite element software, providing sufficient and reliable data support for the subsequent training and testing of deep network models.

#### 2.3.1. Thermal Analysis

Based on the basic theory of the heat conduction differential equation, combined with finite element birth and death technology, a three-dimensional finite element analysis model coupled with a moving heat source was established to carry out transient heat transfer simulation for the welding process of large flange shafts so as to accurately obtain the temperature field distribution and heat transfer law in the welded joint area. Among them, during the welding heat transfer process, the heat transfer in the welded part is obtained as follows:

(1) $$\frac{\partial }{\partial x}\left(k\frac{\partial T}{\partial x}\right)+\frac{\partial }{\partial y}\left(k\frac{\partial T}{\partial y}\right)+\frac{\partial }{\partial z}\left(k\frac{\partial T}{\partial z}\right)+Q\left(x,y,z,t\right)=\rho c_{p}\frac{\partial T}{\partial t}$$

where ρ is the density of the material; $c_{p}$ is the specific heat capacity of the material; k is the thermal conductivity of the material; x,y,z are the coordinates; $Q\left(x,y,z,t\right)$ is the internal heat generation rate; and T,t are the current temperature and time of the material, respectively.

In this study, it is assumed that only convection and radiation between the outer surface of the entire finite element model and the surrounding air are considered. The solution to the transient heat transfer in the finite element analysis (Equation (2)) also requires specific initial conditions, including the initial temperature and the boundary conditions for temperature transfer [34].

(2) $$k\frac{\partial T}{\partial n}=q-h_{c}\left(T-T_{s}\right)-\varepsilon \sigma \left(T-T_{s}\right)$$

where $h_{c},\varepsilon ,\sigma ,T_{s}$ are the convective heat transfer coefficient, radiant emissivity coefficient, Stefan–Boltzmann constant and ambient temperature of the material, respectively, and the ambient temperature is assumed to be 25 °C.

**Figure 4:**
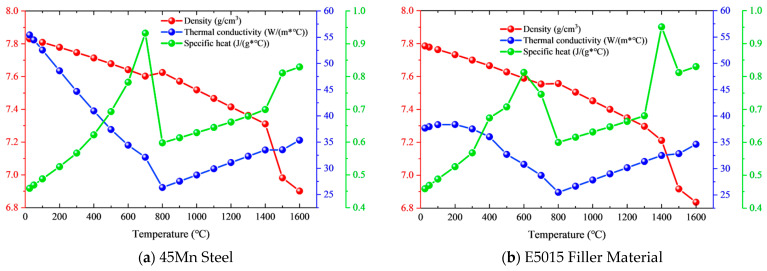
Thermal property curves of 45Mn steel and E5015 filler material.

According to Ref. [29], the thermal properties of 45Mn steel and E5015 filler material are shown in Figure 4.

#### 2.3.2. Heat Source Model

**Figure 5:**
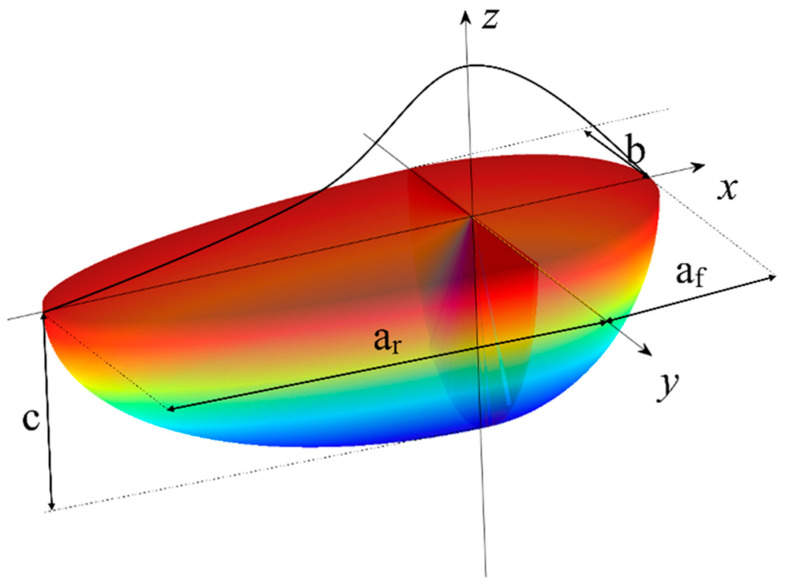
Goldak double-ellipsoidal moving heat source model.

According to actual welding tests, the temperature gradient before and after the heat source exhibits an asymmetric distribution, and the temperature gradient at the front of the heat source becomes significantly steeper. The Goldak double-ellipsoidal moving heat source model [35] was used to simulate the heat flux density of the volumetric welding heat source, as shown in Figure 5. In this analysis, the double-ellipsoidal heat source composed of two ellipsoids moves forward along the *x*-axis. The expressions for the heat flux density in the front and rear parts of the heat source are given as follows:

(3) $$q_{f}\left(x,y,z\right)=\frac{6\sqrt{3}f_{f}Q}{\pi \sqrt{\pi }a_{f}bc}e^{\left[-\left(\frac{3x^{2}}{a_{f}^{2}}+\frac{3y^{2}}{b^{2}}+\frac{3z^{2}}{c^{2}}\right)\right]}$$

(4) $$q_{r}\left(x,y,z\right)=\frac{6\sqrt{3}f_{r}Q}{\pi \sqrt{\pi }a_{r}bc}e^{\left[-\left(\frac{3x^{2}}{a_{r}^{2}}+\frac{3y^{2}}{b^{2}}+\frac{3z^{2}}{c^{2}}\right)\right]}$$

(5) Q=ηUI

where $q_{f}\left(x,y,z\right)$ and $q_{r}\left(x,y,z\right)$ are the heat flux densities in the front and rear parts of the welding zone, respectively; Q is the heat source power; η is the welding thermal efficiency, U is the welding arc voltage, and I is the welding current; $f_{f},f_{r}$ are the front and rear energy distribution parameters ($f_{f}+f_{r}=2$), $a_{f},a_{r}$ are the lengths of the front and rear semi-axes in the double-ellipsoidal moving heat source model, and b,c are the parameters of the moving volumetric heat source, respectively.

#### 2.3.3. Mechanical Analysis

In the finite element analysis, the total strain increment at each material point can be expressed using the following formula:

(6) $$\Delta \varepsilon_{\mathrm{total}}=\Delta \varepsilon_{\mathrm{elastic}}+\Delta \varepsilon_{\mathrm{plastic}}+\Delta \varepsilon_{\mathrm{thermal}}+\Delta \varepsilon_{\mathrm{creep}}+\Delta \varepsilon_{\mathrm{phase}}$$

where $\Delta \varepsilon_{\mathrm{total}},\Delta \varepsilon_{\mathrm{elastic}},\Delta \varepsilon_{\mathrm{plastic}},\Delta \varepsilon_{\mathrm{thermal}},\Delta \varepsilon_{\mathrm{creep}},\Delta \varepsilon_{\mathrm{phase}}$ are the total strain increment, elastic strain increment, plastic strain increment, thermal strain increment, creep strain increment and transformation strain increment, respectively. Nevertheless, owing to the short duration of the welding thermal cycle, the contribution of creep deformation to the macroscopic residual stress and distortion is insignificant and can be safely neglected in the present model. Likewise, phase transformation-induced strain is disregarded, as no evident phase transformation takes place in 45Mn steel under the applied welding conditions. Therefore, the total strain increment at each material point can be simplified as follows:

(7) $$\Delta \varepsilon_{\mathrm{total}}=\Delta \varepsilon_{\mathrm{elastic}}+\Delta \varepsilon_{\mathrm{plastic}}+\Delta \varepsilon_{\mathrm{thermal}}$$

(8) $$\varepsilon_{\mathrm{thermal}}=k\cdot \Delta T$$

**Figure 6:**
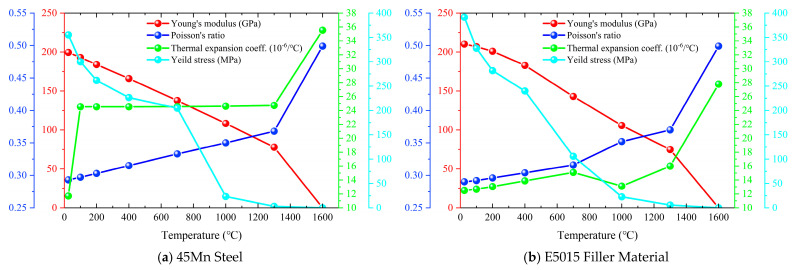
Physical property curves of 45Mn steel and E5015 filler material.

Isotropic Hooke’s law was used to model the elastic behavior, relying on the temperature-dependent Young’s modulus and Poisson’s ratio. In addition, the thermal strain in the mechanical simulation depends on the coefficient of thermal expansion. Furthermore, the yield strength in the mechanical calculation characterizes the plastic behavior of the material. According to Ref. [29], the physical properties of 45Mn steel and E5015 filler material are shown in Figure 6.

#### 2.3.4. Finite Element Simulation

**Figure 7:**
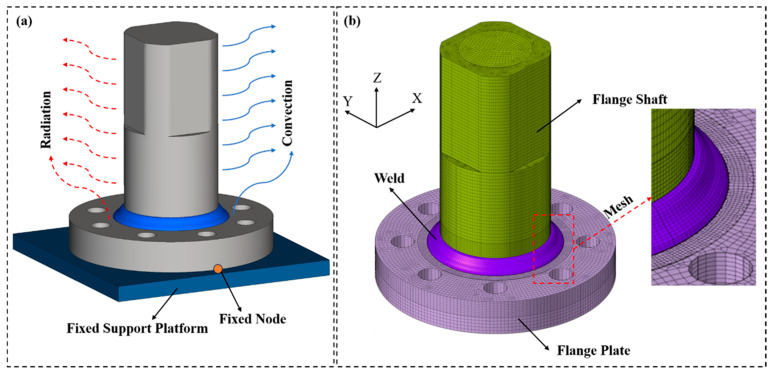
Boundary conditions and meshing of the finite element model: (**a**) boundary conditions; (**b**) meshing.

To avoid secondary residual stress in the welded component caused by excessive constraints, strict control was applied to the boundary conditions of the finite element model in this study. Displacement and rotation constraints were imposed on selected nodes on the bottom surface of the flange plate, and node constraints were also applied to the contact surface between the flange shaft and the flange plate, ensuring that their relative positions remained constant during welding. The detailed boundary conditions are shown in Figure 7a.

A partitioned strategy combining refined and coarse meshing was adopted for the model meshing. Sweep meshing technology and advanced partitioning algorithms were integrated to construct hexahedral meshes for the base metal and weld zone, balancing computational accuracy and solving efficiency. Key regions such as the weld and heat-affected zone were meshed finely with an element size of 1 mm. By densifying the elements, the gradient variations in the temperature field, stress field, and microstructure evolution were captured accurately. The base metal region far from the weld was meshed coarsely with an element size of 2 mm to improve solving efficiency while meeting accuracy requirements, ensuring the regularity and coordination of the overall mesh [29]. The final hexahedral mesh model of the welded component is shown in Figure 7b.

The finite element analysis simulated the complete welding process through four types of interactions: the deactivation of birth–death elements, thermal convection heat transfer, thermal radiation heat transfer, and the reactivation of birth–death elements. The specific simulation procedure is as follows: in the initial analysis step, the birth–death elements in the weld zone were first set to the deactivated state. Meanwhile, surface heat exchange between the weldment and the surrounding environment was defined, with a thermal convection coefficient of 25 W/(m^2^·°C), and thermal radiation between the weldment and the external environment was considered with an emissivity of 0.8. In the cooling stage, the activation and state switching of the remaining weld elements were completed through the interaction of birth–death element deactivation, thereby simulating the full completion of the welding process.

### 2.4. Result Extraction and Processing

After completion of the finite element simulation, systematic data extraction was performed on the key physical field responses during the welding process based on the post-processing module. The extracted indicators and corresponding rules are as follows: For the temperature field, the peak temperature of the welding thermal cycle was extracted at the weld center region, and the base metal peak temperature was extracted at the base metal reference region, with data collected at the simulation instant when the thermal cycle of each region reached its temperature peak. For structural deformation, the X-direction axial deformation and Y-direction radial deformation at key nodes on the central axis of the flange shaft were extracted after the welding process was completed and cooled to room temperature (25 °C). For residual stress, the peak equivalent stress of elements on the weld section and on the bonding surface between the flange shaft and flange plate was extracted. All peak indicators were taken as the statistical maximum values of elements/nodes within the target region. All peak indicators are defined as the maximum values among all element/node results within the target monitoring region, used to characterize the extreme responses at the most critical locations during welding. During data extraction, outliers are identified based on the numerical continuity of adjacent elements/nodes. If the result of a single element or node deviates significantly from the mean value of the surrounding area and lacks physical rationality, it is identified as an outlier caused by numerical oscillation and removed. This ensures that the extracted data truly reflect the distribution of the welding physical fields. By collecting the above indicator data for each working condition, a dataset of temperature, deformation, and residual stress for large flange shaft arc welding containing 100 groups of samples was finally constructed.

**Figure 8:**
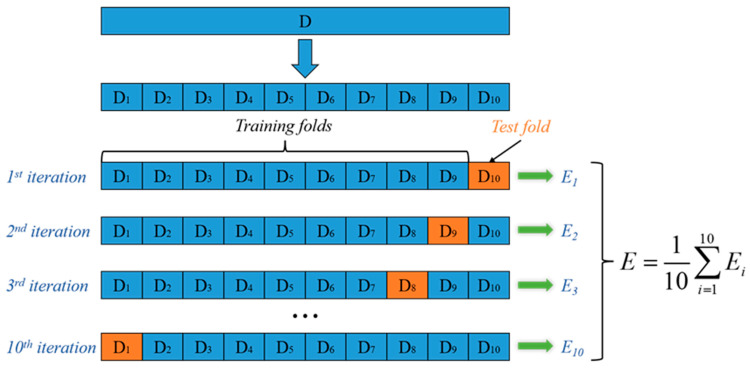
Schematic of the 10-fold cross-validation method used in the welding simulation data for large flange shafts.

Considering the relatively limited sample size of the dataset, the direct adoption of a fixed train–test split tends to cause model overfitting and reduce the generalization ability of the results. To avoid the limitations caused by fixed data partitioning, the k-fold cross-validation method was introduced in this study for model evaluation [36]. First, all 100 standardized samples were randomly shuffled to eliminate potential bias introduced by the original sample order. The samples were then equally divided into 10 folds, each containing 10 independent samples. The choice of k = 10 is motivated by the nature of the present 100-sample medium-sized dataset. Using 10-fold cross-validation allows 90% of the data to be used for training in each iteration, with only 10% held out for testing. This strategy maximizes the use of limited data, effectively reduces the risk of overfitting, and ensures stable and reliable model evaluation. During model training and validation, a single fold was sequentially used as the test set, and the remaining nine folds were combined as the training set to complete iterative model training and performance testing. The above process was repeated until all folds had completed the role rotation of the test set. Taking the Mean Absolute Error (MAE) as a core evaluation metric, the average value of 10 validation results was calculated as the final model performance, which effectively reduces the risk of small-sample overfitting and improves the generalization ability and evaluation stability of the subsequent welding parameter–physical response prediction model. The 10-fold cross-validation procedure adopted in this study is shown in Figure 8, and the definition of the core evaluation indicator is given as follows:

(9) $$MAE=\frac{1}{n}\sum_{i=1}^{n}\left|{\hat{y}}_{i}-y_{i}\right|$$

where ${\hat{y}}_{i}$ denotes the model predicted value, y denotes the true value, and n denotes the number of samples.

## 3. Construction of Welding Prediction Model

To achieve an accurate prediction of the welding performance (peak temperature, deformation, residual stress, etc.) based on welding process parameters, it is necessary to construct an appropriate prediction model. In this section, typical models from traditional machine learning (XGBoost, RF, RBF-SVR, etc.) and deep learning (MLP, TabNet, FT-Transformer, etc.) are selected respectively, and six prediction models are constructed for subsequent training, validation, and comparison.

### 3.1. Machine Learning Models

#### 3.1.1. XGBoost Model

XGBoost (Extreme Gradient Boosting) [37,38,39] is a machine learning algorithm based on the gradient boosting framework. By introducing techniques such as regularization, parallel computing, and pruning, it significantly improves the performance of the traditional Gradient Boosting Decision Tree (GBDT) and has become one of the most efficient ensemble learning algorithms to date. XGBoost is suitable for large-scale datasets and is widely applied to tasks such as classification, regression, and ranking.

The objective of XGBoost is to minimize the following loss function:

(10) $$L\left(\theta \right)=\sum_{i=1}^{n}l\left(y_{i},{\hat{y}}_{i}\right)+\sum_{k=1}^{K}\Omega \left(f_{k}\right)$$

where $l\left(y_{i},{\hat{y}}_{i}\right)$ is the loss function for the *i*-th sample, and common loss functions include log loss and mean squared error (MSE); $f_{k}$ denotes the *k*-th tree; and $\Omega \left(f_{k}\right)$ is the regularization term that controls the complexity of the tree, which is usually expressed as

(11) $$\Omega \left(f_{k}\right)=\gamma T+\frac{1}{2}\lambda \sum_{j=1}^{T}w_{i}^{2}$$

where *T* is the number of leaf nodes of the tree, *λ* is the regularization parameter, and *w_i_* is the weight of each leaf node in the tree. The introduction of the regularization term is to prevent overfitting and control the complexity of the model. XGBoost adopts the gradient boosting method and optimizes the current prediction by minimizing the loss function. The current model is defined as

(12) $${\hat{y}}_{i}^{\left(t\right)}=\sum_{k=1}^{t}f_{k}\left(x_{i}\right)$$

**Figure 9:**
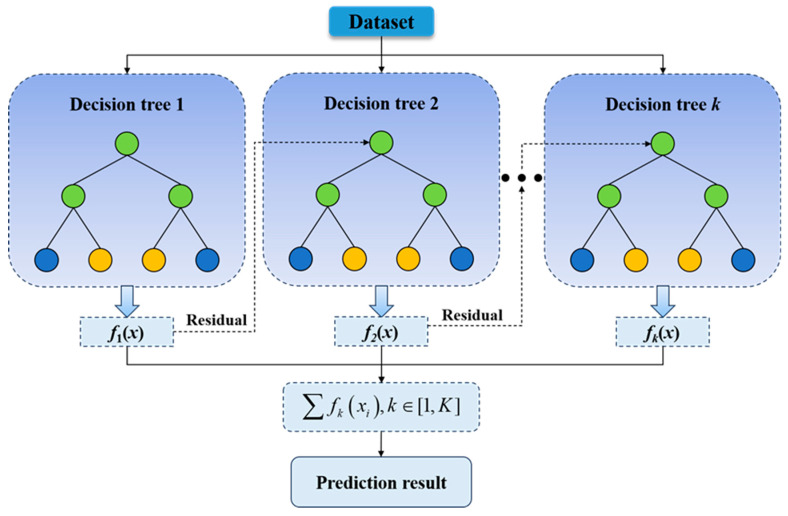
Flowchart of the XGBoost algorithm.

Then, the residual is obtained by calculating the negative gradient of the current model, and a new tree model is fitted on this residual. The training objective of each tree is to minimize the objective function via gradient descent. The workflow of the XGBoost algorithm is shown in Figure 9.

#### 3.1.2. RBF-SVR Model

RBF-SVR (Radial Basis Function Support Vector Regression) [40,41,42] is a regression model improved based on Support Vector Machine (SVM). Its core is to map the original input features into a high-dimensional feature space through the radial basis function (RBF) kernel and construct an optimal hyperplane to realize prediction for unknown samples. This model has a strong nonlinear fitting ability and robust performance on small-sample data. It does not rely on assumptions about data distribution and can effectively handle complex nonlinear mapping between welding process parameters and residual stress deformation in welding processes. Its generalization ability is superior to traditional linear regression models.

**Figure 10:**
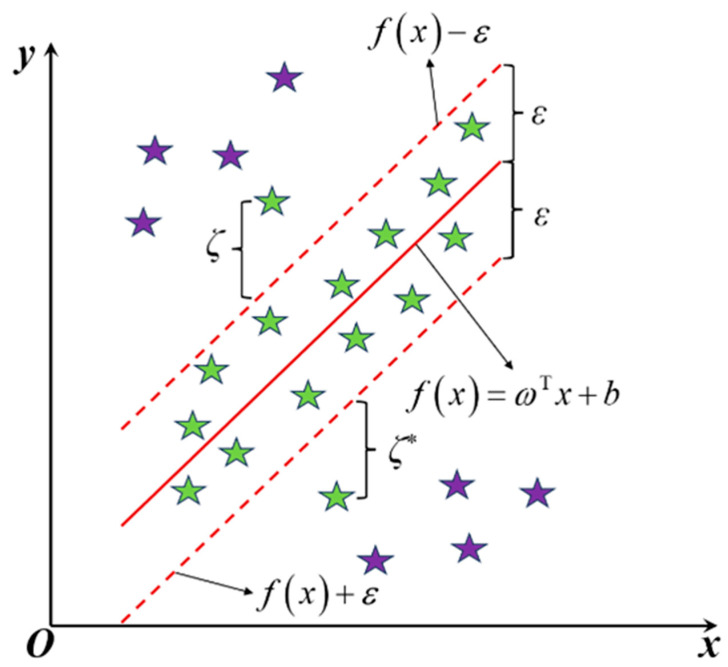
Schematic diagram of the RBF-SVR algorithm principle.

The SVR problem can be described as follows:

(13) $$f\left(x\right)=\omega^{T}x+b$$

(14) $$\underset{\omega ,b,\zeta_{i},{\hat{\zeta }}_{i}}{\min}\frac{1}{2}{\left\|\omega \right\|}^{2}+C\sum_{i=1}^{m}\left(\zeta_{i}+\zeta_{i}^{*}\right)$$

(15) $$\mathrm{Subject}\;\mathrm{to}\;y_{i}-f\left(x_{i}\right)\leq \varepsilon +\zeta_{i},\zeta_{i}\geq 0$$

(16) $$f\left(x_{i}\right)-y_{i}\leq \varepsilon +\zeta_{i}^{*},\zeta_{i}^{*}\geq 0$$

where ω denotes the weight vector, b is the bias term, $\zeta ,\zeta^{*}$ are slack variables, ε is the insensitive loss parameter, and C is the penalty coefficient. The schematic diagram of the RBF-SVR algorithm principle is shown in Figure 10.

#### 3.1.3. Random Forest Model

**Figure 11:**
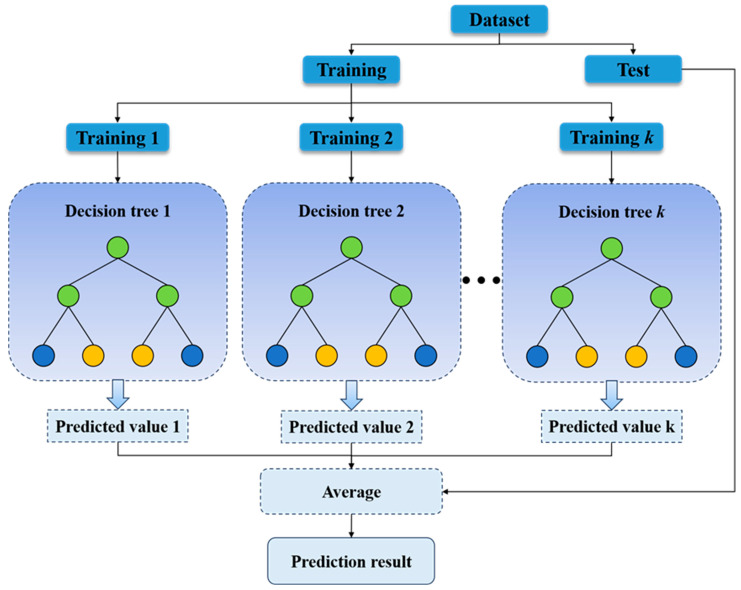
Flowchart of the RF algorithm.

Random Forest (Random Forest) [43,44,45] is an ensemble learning model based on decision trees. Its core idea is to generate multiple training subsets through bootstrap sampling, train individual CART regression trees respectively, and finally output the results by voting (classification) or averaging (regression). The model has a simple structure, fast training speed, and strong anti-overfitting ability. It is insensitive to outliers and missing values and can output feature importance. It can effectively capture the nonlinear relationship between welding process parameters and performance indicators such as residual stress and temperature. In the training process, bootstrap sampling and random feature selection are used to construct each tree, which increases the diversity among trees and reduces the risk of overfitting. It is often used as a baseline model to compare and verify the performance of other models. The workflow of the RF algorithm is shown in Figure 11.

#### 3.1.4. Setting of Model Hyperparameters

The performance of machine learning models strongly depends on the choice of hyperparameters. However, the hyperparameter space is complex, and an exhaustive search is time-consuming, with a high computational cost. Especially under the limited sample size in this study, excessive searching tends to introduce the risk of overfitting. Therefore, the hyperparameter settings for each model in this study were determined based on mature engineering experience and the published literature in related fields, and were further adjusted according to the distribution characteristics of the dataset used in this work [41,46,47]. To ensure training stability and fair evaluation, all models were trained and validated under a unified 10-fold cross-validation framework. The result fluctuations under small-sample conditions were reduced by random shuffling and multi-fold rotation verification. In the preliminary validation stage, each hyperparameter combination was tested through repeated experiments, and the set with a stable performance and good generalization in cross-validation was selected as the final configuration, balancing prediction accuracy and training efficiency. The specific hyperparameter settings are listed in Table 4, which can serve as a benchmark for subsequent performance comparisons among different models.

### 3.2. Deep Learning Models

#### 3.2.1. Multi-Layer Perceptron (MLP) Model

Multi-Layer Perceptron (MLP) [48,49,50] is a typical deep fully connected neural network whose structure consists of an input layer, several hidden layers, and an output layer. Its computation mainly includes forward propagation and backpropagation. The forward propagation process of MLP is calculated layer by layer using the weight matrix W, the bias vector b of each layer, and the input of the corresponding layer [51]. The forward propagation calculation of a specific layer in matrix form is as follows:

(17) $$a^{l}=\sigma \left(z^{l}\right)=\sigma \left(W^{l}a^{l-1}+b^{l}\right)$$

where $a^{l}$ denotes the output of the *l*-th layer, $W^{l}$ denotes the weight coefficient matrix of the *l*-th layer; $a^{l-1}$ is the output of the (*l* − 1)-th layer and also the input of the *l*-th layer, $b^{l}$ is the bias vector of the *l*-th layer, and $z^{l}$ is the linear computation result of the *l*-th layer.

**Figure 12:**
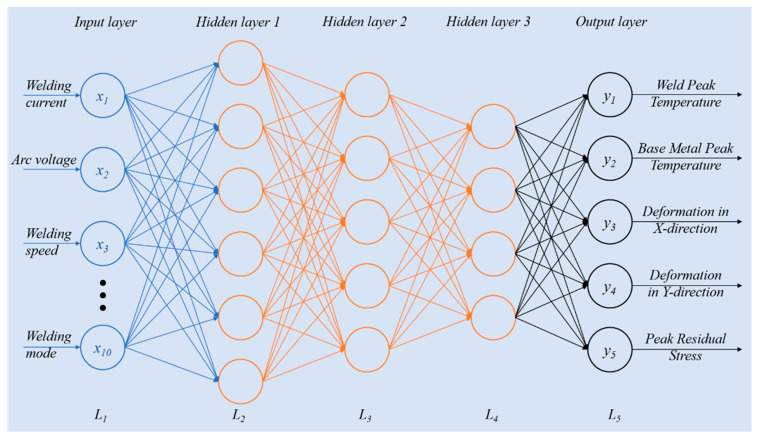
Schematic diagram of the MLP deep learning model structure.

An MLP model refers to a neural network with multiple hidden layers. The structural schematic of the MLP model in this study is shown in Figure 12. A fully connected mode is adopted between layers, that is, all neurons in the *L*-th layer are connected to all neurons in the (*L* + 1)-th layer. Locally, each neuron satisfies a linear relationship, and the output of the neuron is realized through an activation function.

(1) Activation Function

Commonly used activation functions include Sigmoid, tanh, PReLU, and ReLU. Both Sigmoid and tanh suffer from the vanishing gradient problem, which leads to extremely slow updates for model parameters when the network depth is large. The ReLU activation function effectively addresses the vanishing gradient problem and significantly improves the model convergence speed compared with Sigmoid and tanh [36]. Therefore, ReLU was finally selected as the activation function in this study.

(2) Neural Network Structure

Based on the dataset derived from the arc welding simulation results of large flange shafts, a five-layer DNN model was constructed in this study, and its hyperparameters are listed in Table 5. The input layer contains 10 neurons, matching the dimension of the input features after encoding and expansion. The original input parameters are five (current, voltage, welding speed, torch incidence angle, and welding mode). Among them, current, voltage, welding speed, and torch incidence angle are continuous features, directly used as four basic dimensions. The welding mode is a binary variable (continuous welding, skip welding), which is expanded to two dimensions through one-hot encoding. Combined with four hidden correlation dimensions reserved for feature interaction, a 10-dimensional input feature vector is finally formed to ensure that the model can fully capture the complex nonlinear relationships among parameters. The output layer contains five neurons, consistent with the feature dimensions of the five prediction targets (weld peak temperature, base metal peak temperature, X-direction deformation, Y-direction deformation, and peak residual stress). Given the characteristics of the 100-sample small dataset in this study, the structural design of the five-layer MLP model is clearly reasonable. A moderate number of network layers and neurons is adopted, which avoids overfitting caused by an overly complex structure while ensuring the model’s ability to capture the complex nonlinear relationships between welding process parameters and multi-physics fields, making it well suited for small-sample training scenarios.

According to the actual training performance, the number of iterations was set to 600 to ensure the sufficient convergence of the model. A batch size of 3 was adopted in the training process, which adapts to the small dataset scale of 100 samples and ensures the stability of parameter updates. The learning rate was set to 0.0001, which can effectively avoid excessive gradient oscillation at the early training stage and ensure an accurate convergence to the optimal solution in the later stage. Furthermore, to further mitigate overfitting under small-sample conditions, early stopping was additionally introduced during training. The validation MAE was used as the monitoring metric: if the validation MAE did not decrease for 15 consecutive epochs, training was automatically terminated and the optimal parameters were saved. This prevents the model from overfitting the training data and preserves its generalization ability. Together with the batch partitioning strategy, it achieves stable training and ensures no significant overfitting in the final model.

#### 3.2.2. TabNet Model

TabNet [52,53,54] is a deep learning model designed specifically for tabular data. Traditional deep learning methods such as CNN and RNN are mainly applied to structured data like images, speech, and text, and TabNet is proposed to fill this gap. It can efficiently process tabular data and performs particularly well in classification and regression tasks on structured data. Through a novel self-attention mechanism, TabNet combines the advantages of feature selection and deep neural networks (DNNs), enabling efficient learning without explicit feature engineering.

The core idea of TabNet is to select important features via a self-attention mechanism and guide the learning process of the model. Different from traditional neural networks, the most distinctive feature of TabNet is its introduction of interpretability and flexibility: it can automatically select and combine input features, thus effectively improving performance. The model achieves both the strong fitting ability of deep learning and the interpretability of traditional machine learning. It can automatically learn the importance weights of welding process parameters without manual feature selection, making it suitable for welding performance prediction tasks with large sample sizes and complex feature interactions. Its prediction accuracy is generally superior to traditional MLP models, and it is well adapted to one-dimensional tabular data.

**Figure 13:**
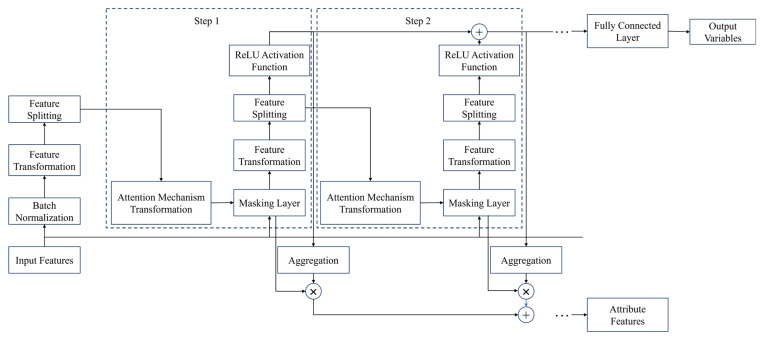
Schematic diagram of TabNet decoder dtructure.

The TabNet model mainly consists of two components: a feature selection module and a feature processing module. The feature selection module adaptively screens key features in tabular data using an attention mechanism, effectively reducing the input feature dimension while passing the selected features to the subsequent feature processing module. The feature processing module gradually processes the selected features through multi-step decision operations to accomplish the prediction of the target variable. This process is similar in form to the node splitting procedure of decision trees; however, TabNet continuously mines and utilizes the intrinsic relationships among features for iterative optimization, thereby significantly improving prediction accuracy. Figure 13 shows the encoder structure of TabNet, whose core components include the feature transformer, attention transformer, and mask layer.

Considering the characteristics of the 100-sample small dataset in this study, the structural design of the TabNet model, especially key parameters such as the number of attention iteration steps, has been specifically optimized. Three attention iteration steps are employed, which not only avoids excessive steps that would cause parameter redundancy and increase overfitting risk, but also ensures that the attention mechanism can accurately select critical welding process parameters. This balances feature extraction accuracy and training efficiency and effectively satisfies the feature learning requirements under small-sample conditions.

#### 3.2.3. FT-Transformer Model

FT-Transformer (Feature Token Transformer) [55,56,57] is a deep learning model designed for tabular data, which combines the powerful capability of the Transformer model with the characteristics of tabular data. Traditional Transformer models were originally developed for natural language processing (NLP) tasks and excel at processing sequential data. However, their performance is often unsatisfactory when directly applied to tabular data. FT-Transformer introduces the “Feature Token” and other innovative designs, enabling the Transformer to effectively process tabular data and achieve outstanding performance in many tabular data tasks. The key of the FT-Transformer model lies in its feature representation method and the improved Transformer structure, which allow it to handle datasets with high-dimensional features.

**Figure 14:**
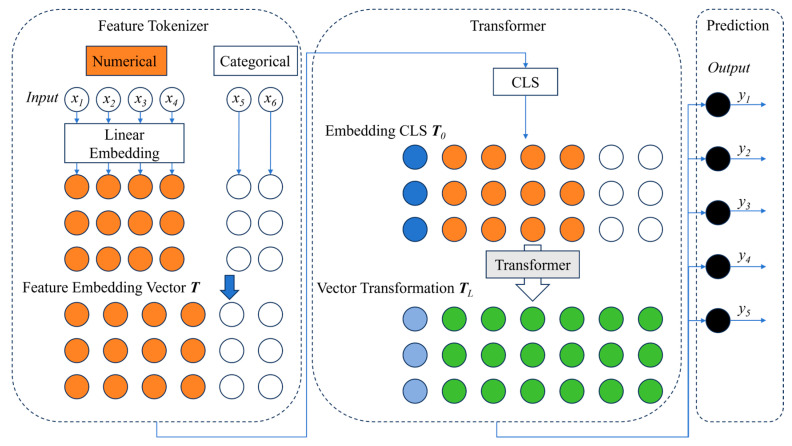
Schematic diagram of FT-Transformer deep learning model architecture for welding process parameter prediction.

The core idea of the FT-Transformer is to address the unique challenges of tabular data through an innovative feature representation approach combined with the self-attention mechanism of the Transformer. The FT-Transformer model treats tabular data as a set of features rather than conventional sequential input and processes these feature tokens as part of the input to the Transformer. The schematic diagram of the FT-Transformer model used in this study is shown in Figure 14.

For the 100-sample small-sample scenario in this study, the FT-Transformer adopts a lightweight architecture to avoid overfitting caused by the complex structure of conventional Transformers. Specifically, only one Transformer encoder layer and two attention heads are used, with the feature embedding dimension and feed-forward network dimension carefully controlled. While retaining the self-attention mechanism and the ability to capture feature interactions, the model complexity is minimized to fit small-sample training. In this way, a good balance between fitting capacity and generalization performance is achieved.

#### 3.2.4. Setting of Model Hyperparameters

Based on the dataset obtained from the arc welding simulation of large flange shafts, the TabNet model and the FT-Transformer model were constructed in this study. Both models are suitable for one-dimensional tabular welding process data, and their hyperparameter settings are listed in Table 6. The inputs of both models contain 10 feature dimensions, matching the dimension of the welding process parameters after encoding and expansion. The original input parameters include five variables: current, voltage, speed, torch incidence angle, and welding mode. Among them, current, voltage, speed, and torch incidence angle are continuous features, directly used as four basic dimensions. Welding mode is a binary variable (continuous welding, skip welding), which is expanded into two dimensions through one-hot encoding. Combined with the four hidden interaction dimensions reserved for feature interaction, a 10-dimensional input feature vector is finally formed to ensure that the model can fully capture the complex nonlinear relationships among welding process parameters. The output layer contains five neurons, corresponding to the five welding performance prediction targets: peak weld temperature, peak base metal temperature, X-direction deformation, Y-direction deformation, and peak residual stress.

For the TabNet model, considering the characteristics of the small-sample dataset, the decision dimension n_d and the attention dimension n_a are both set to 8, and the number of attention steps n_steps is set to 3, balancing feature selection accuracy and training efficiency. The sparse regularization coefficient γ is set to 1.3 to enhance the feature discrimination ability of the attention mechanism. The numbers of independent decision layers and shared decision layers are both set to 2 to capture the interactions among features of different dimensions. During training, the learning rate is set to 0.02, the batch size is set to 8 to adapt to the small dataset of 100 samples, and the maximum number of training epochs max_epochs is set to 150, which ensures sufficient convergence and avoids overfitting. Meanwhile, random_state = 42 is set to ensure experimental reproducibility. During training, early stopping was synchronously integrated to mitigate overfitting in collaboration with dropout regularization and mini-batch training. This strategy further enhanced the model’s generalization ability and ensured stable performance under small-sample conditions.

For the FT-Transformer model, a lightweight structure is adopted to suit the small-sample scenario. The feature embedding dimension d_model is set to 16, the number of attention heads n_head is set to 2, and the number of Transformer encoder layers is set to 1 to avoid overfitting caused by excessive model complexity. The feed-forward network dimension dim_feedforward is set to 32 to enhance the feature fusion ability, and the dropout probability is set to 0.1 to further suppress overfitting. The Adam optimizer is used during training with a learning rate of 0.001 to avoid excessive gradient oscillation at the early stage of training. The batch size is set to 8 and the number of training epochs is set to 150. The mean squared error (MSE) is adopted as the loss function, which is suitable for multi-output regression tasks and ensures the accurate prediction of welding performance indicators. random_state = 42 is also set to ensure repeatable experimental results. During training, an early stopping strategy was introduced, with the validation set MAE as the monitoring metric to prevent over-training. This, in combination with dropout regularization, forms a comprehensive anti-overfitting system that is well-suited for small-sample training scenarios.

The hyperparameters of all models in this study were selected using a two-step approach: initial range determination via a literature review, followed by optimal selection through 10-fold cross-validation. First, reasonable candidate ranges for key hyperparameters (e.g., learning rate, batch size, hidden layer dimensions) were determined based on the relevant literature and common practices in the field of welding prediction. Then, 10-fold cross-validation was performed, with the test set Mean Absolute Error (MAE) as the evaluation metric, to select the hyperparameter combination with the best generalization performance, ensuring the scientific reliability of the parameter settings.

## 4. Results and Discussion

### 4.1. Comparison of Model Performance

To evaluate the performance of different models in the multi-physics prediction of large flange shaft welding, this section takes the Mean Absolute Error (MAE) as the evaluation index. Based on the error fluctuation characteristics, prediction residual distributions, and performances on extreme samples evaluated via 10-fold cross-validation, a comprehensive comparative analysis was conducted across six models—MLP, TabNet, FT-Transformer, XGBoost, RBF-SVR, and RF—assessing their prediction accuracy, generalization capability, and robustness across three key dimensions: temperature, deformation, and residual stress. Considering that the dataset consists of only 100 finite element simulation samples, placing it in the small-sample category for deep learning applications, 10-fold cross-validation was employed to ensure the statistical reliability of the results. The minimal fluctuations in validation errors across the ten folds provide a scientific basis for model selection and optimization.

#### 4.1.1. Temperature

**Figure 15:**
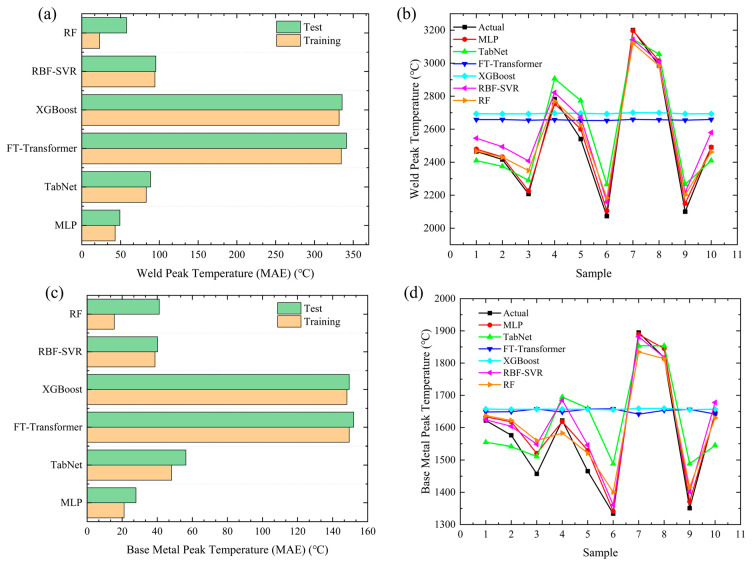
Comparison of welding peak temperature prediction MAE using different models and sample-level prediction result validation: (**a**) MAE of weld peak temperature; (**b**) prediction of weld peak temperature; (**c**) MAE of base metal peak temperature; (**d**) prediction of base metal peak temperature.

Temperature is a key physical quantity characterizing the welding thermal cycle process, which directly affects the microstructure evolution and property formation of welded joints. Therefore, the accuracy of temperature prediction is of great significance for the numerical simulation of welding processes. Figure 15 presents the MAE comparison and sample prediction curves of different models in the temperature prediction of large flange shaft welding. Figure 15a,c show the comparison of Mean Absolute Error (MAE) between the training set and test set for each model in the prediction of weld peak temperature and base metal peak temperature, respectively. All models were evaluated using 10-fold cross-validation, and the variation in test MAE across folds was less than 8%, indicating stable prediction performance, even under small-sample conditions. From the overall trend, the training set MAE of all models is lower than the test set MAE, indicating that each model has achieved effective fitting during the training process without obvious overfitting, and the generalization performance is relatively stable. For peak temperature prediction, the test set MAE values of FT-Transformer and XGBoost are significantly higher than those of the other models, indicating that their prediction accuracy under the small-sample setting in this study is much lower and that these two models perform poorly. The test errors of RBF-SVR and TabNet are similar, at a medium level; the test set MAE of MLP and RF is the smallest. However, for the RF model, the difference between the training set and test set MAE is relatively large, so its prediction performance is inferior to that of the MLP model.

Figure 15b,d show the comparison curves between the sample-level predicted values and the true values of each model for the weld peak temperature and base metal peak temperature, respectively. From the overall prediction trend, all models can effectively fit the temperature variation law of most samples, and the prediction curves are generally consistent with the variation trend of the true values. At a few extreme sample points, some models show varying degrees of prediction deviation. Among them, the predicted values of FT-Transformer and XGBoost deviate significantly from the true values, while the prediction curve of the MLP model fits the true values best, demonstrating a more stable prediction performance. It can be seen that the MLP model has a better reliability and accuracy in the fine sample-by-sample prediction of the welding temperature field.

#### 4.1.2. Deformation

**Figure 16:**
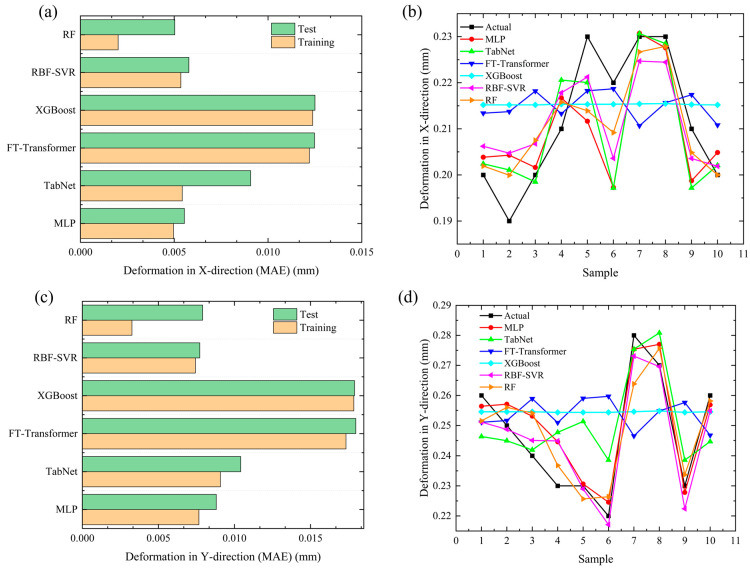
Comparison of welding deformation prediction MAE using different models and sample-level prediction result validation: (**a**) MAE of deformation in X-direction; (**b**) prediction of deformation in X-direction; (**c**) MAE of deformation in Y-direction; (**d**) prediction of deformation in Y-direction.

Welding deformation directly affects the dimensional accuracy and assembly performance of components and is a key indicator that needs to be controlled in the welding process. Figure 16a,c show the Mean Absolute Error (MAE) of the training set and test set for each model in X-direction deformation and Y-direction deformation prediction, respectively. Figure 16b,d show the corresponding comparison curves between the sample-level predicted values and the true values of each model for X-direction and Y-direction deformation. From the MAE comparison results (Figure 16a,c), the training set MAE of all models is lower than the test set MAE, indicating that each model has achieved effective fitting during training without obvious overfitting, and the generalization performance is reliable. In both X-direction and Y-direction deformation prediction, the error distribution of each model is highly consistent: the MLP model achieves the smallest test set MAE and the highest prediction accuracy; the RF model shows a slightly higher test error than MLP, ranking second; the test errors of RBF-SVR and TabNet are at a medium level, while the test set MAE of XGBoost and FT-Transformer is obviously higher, with a larger prediction deviation and the worst prediction accuracy.

From the sample-level prediction curves (Figure 16b,d), all models can effectively fit the deformation variation law of most samples, and the prediction curves are generally consistent with the trend of the true values, which further verifies the effectiveness of model fitting. Prediction robustness is comprehensively assessed using error distribution, linear correlation between predictions and references, and deviation magnitude. Among them, the MLP model has the highest fitting degree with the true values and the smallest fluctuation, showing a stable and reliable sample-by-sample prediction performance, which is consistent with the conclusion that its test set MAE is the smallest in Figure 16a,c. The RF model takes second place in prediction accuracy, with small deviations from the true values. The prediction curves of RBF-SVR and TabNet show certain fluctuations with medium-level deviations, corresponding to their medium MAE values. In contrast, the predicted values of XGBoost and FT-Transformer deviate significantly from the true values, especially at a few extreme sample points. This result fully corresponds to their highest test set MAE and worst prediction accuracy in Figure 16a,c, indicating that their nonlinear mapping ability for the welding deformation field is relatively weak and has difficulty accurately capturing the deformation characteristics of some samples.

#### 4.1.3. Residual Stress

**Figure 17:**
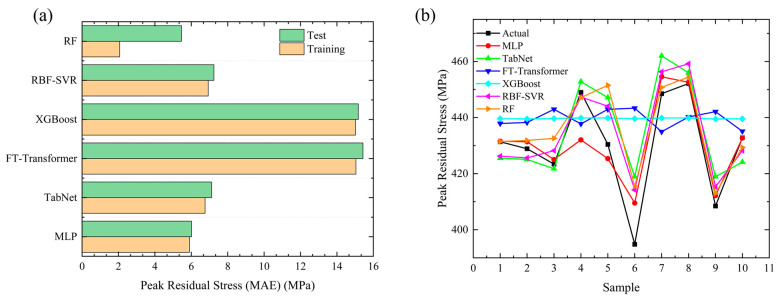
Comparison of welding peak residual stress MAE using different models and sample-level prediction result validation: (**a**) MAE; (**b**) prediction.

Welding residual stress is a key factor affecting the fatigue performance of welded joints and the service life of structures. Its accurate prediction is of great engineering significance for ensuring the reliability of welded structures. Figure 17a shows the comparison of Mean Absolute Error (MAE) between the training set and test set for each model in peak residual stress prediction, and Figure 17b shows the comparison curves between the sample-level predicted values and the true values of each model for peak residual stress. Combining the MAE index and the sample prediction curves, the residual stress prediction performance of each model is analyzed as follows. First, from the MAE comparison results (Figure 17a), the training set MAE of all models is lower than the test set MAE, indicating that each model has achieved effective fitting during the training process without obvious overfitting, and the generalization performance is reliable. In peak residual stress prediction, the MLP model achieves the smallest test set MAE, about 6 MPa, with the highest prediction accuracy. The test set MAE of the RF model is about 6.5 MPa, ranking second. The test set MAE values of RBF-SVR and TabNet are about 7 MPa and 7.2 MPa respectively, at a medium level. In contrast, the test set MAE values of XGBoost and FT-Transformer are significantly higher, about 15 MPa and 15.5 MPa, respectively, showing large prediction deviations and the worst accuracy.

Secondly, from the sample-level prediction curves (Figure 17b), all models can effectively fit the variation law of residual stress for most samples, and the prediction curves are generally consistent with the trend of the true values, which further verifies the effectiveness of model fitting. Among them, the MLP model has the highest fitting degree with the true values, and the prediction deviation at most sample points is less than 5 MPa, showing a stable and reliable sample-by-sample prediction performance, which is consistent with the conclusion that its test set MAE is the smallest in Figure 17a. The RF model ranks second in prediction accuracy, with deviations from the true values mostly within 8 MPa. The prediction curves of RBF-SVR and TabNet show certain fluctuations, with a maximum deviation of about 10 MPa, corresponding to their medium MAE values. In contrast, the predicted values of XGBoost and FT-Transformer deviate significantly from the true values, especially at extreme sample points such as sample 6 and 9, where the deviation can exceed 20 MPa. This result fully corresponds to their highest test set MAE and worst prediction accuracy in Figure 17a, indicating that their nonlinear mapping ability for the welding residual stress field is relatively weak and has difficulty accurately capturing the residual stress characteristics of some samples.

Finally, overall, for the prediction of peak welding residual stress, the MLP model achieves the best overall performance, featuring a high accuracy, low fluctuation, and strong robustness. The RF model ranks second, with advantages in interpretability and training efficiency. RBF-SVR and TabNet perform at a medium level and can meet basic prediction requirements. In contrast, XGBoost and FT-Transformer exhibit a relatively poor prediction accuracy and stability under the small-sample data constraint and task setting of this study. All the above conclusions are based on validation using 100 finite element simulation samples. The performance of these models under extreme welding conditions still needs to be further verified by expanding the dataset in future work.

**Figure 18:**
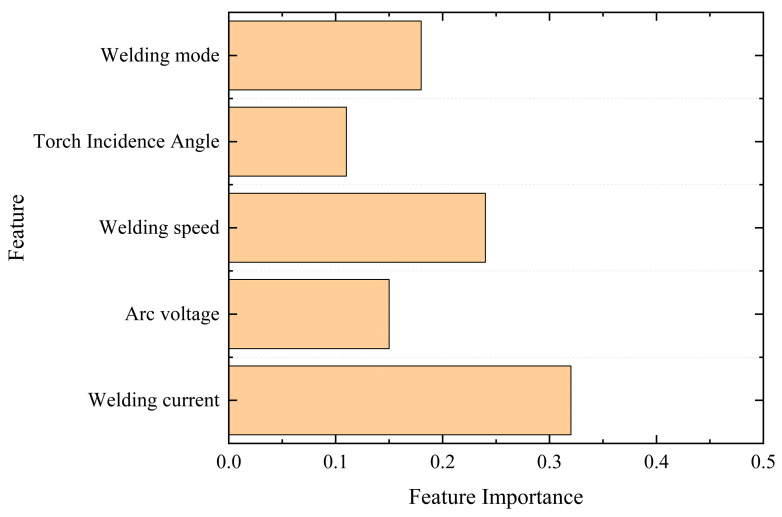
Feature importance of welding process parameters based on Random Forest.

In addition, the Random Forest (RF) model performs favorably in this study, with a prediction accuracy second only to the MLP model, while offering excellent interpretability. To further analyze the influence of process parameters on the welding multi-physical fields, the feature importance of each input parameter was calculated based on the Random Forest model, as illustrated in Figure 18. The results show that the welding current, welding speed, and welding method are the dominant factors affecting the peak temperature, deformation, and residual stress of the weld. Among them, the welding current has the most significant influence on the heat input and peak temperature. The welding speed affects the evolution of deformation and residual stress by altering the heat input duration, and the welding method determines the distribution pattern of the thermal cycle. These observations are consistent with the physical mechanism of welding thermal–mechanical coupling, indicating that the model not only achieves a high prediction accuracy but also captures the physical laws of the actual welding process, which further enhances the engineering practical value of this study.

### 4.2. Validation of MLP Deep Learning Model Predictions

To verify the prediction performance and engineering practicability of the MLP model selected through comparison in Section 4.1, this study designed four validation samples with typical process parameters based on the thermal–mechanical coupling finite element simulation of welding. Among them, samples 1 and 2 adopt the continuous welding mode (welding mode = 1), and samples 3 and 4 adopt the skip welding mode (welding mode = 0). The parameter ranges cover common working conditions in actual production, and the detailed process parameters are listed in Table 7.

The true physical field data (weld peak temperature, base metal peak temperature, X/Y-direction deformation, and peak residual stress) of the four groups of samples were obtained through finite element simulation and used as the “actual values” to compare with and verify the prediction results of the MLP model. The detailed results are listed in Table 8.

It can be seen from the comparison results that the predicted values of the MLP model on all validation samples are in good agreement with the true values from finite element simulation. For thermal indicators such as the weld peak temperature and base metal peak temperature, the prediction errors are all less than 5%. For mechanical indicators including the X/Y-direction deformation and peak residual stress, the prediction errors are controlled within 10%. These results not only verify the prediction accuracy of the MLP model but also demonstrate its generalization ability under different welding modes, providing reliable technical support for the subsequent optimization of welding process parameters. To further verify the physical rationality of the model, a qualitative analysis of the prediction results was conducted, focusing on whether the trends between welding parameters and physical fields conform to physical laws. The results show that the peak weld temperature rises significantly with an increasing welding current. A decrease in welding speed prolongs the heat input duration, which in turn increases axial deformation and residual stress. The temperature field distribution and stress evolution under different welding methods also agree with the thermal–mechanical coupling mechanism. These qualitative trends confirm that the mapping relationship learned by the model is physically consistent, rather than mere data fitting.

However, it should be noted that this study only employed four validation samples for a preliminary generalization test. Due to the limited sample size, the current validation results only reflect the prediction accuracy of the models within the typical working condition range and cannot fully cover extreme working conditions and boundary conditions across the entire parameter space. The generalization ability of the models in untested regions still requires further verification. Meanwhile, this study has not yet performed uncertainty quantification on the prediction results, and there exists a potential risk of increased prediction bias under extreme parameter conditions, which represents a key direction for improvement in future research.

In addition, all training and validation data in this study were derived from experimentally calibrated finite element simulation results, rather than independent experimental measurements. Consequently, the model evaluation results inevitably inherit biases introduced by the approximations and assumptions in the finite element modeling process. Future work will introduce more independent experimental samples, expand the validation dataset, and conduct uncertainty quantification analysis to further improve the reliability and engineering applicability of the models.

## 5. Conclusions

Based on the finite element method (FEM) results obtained from 100 combinations of welding process parameters, this study constructs deep learning models such as MLP for multi-physics field prediction in the welding of large-scale flange shafts. A comparative investigation is conducted against traditional machine learning models, including Random Forest (RF), RBF-SVR, TabNet, XGBoost, and FT-Transformer. It should be emphasized that all conclusions in this paper are derived from FEM simulation data and have not been validated by independent experiments. The dataset of only 100 samples (a relatively small size for deep learning) and the assumptions adopted in FEM modeling may limit the practical engineering generalization ability of the models. Thus, the engineering applicability of the conclusions needs to be further verified through experiments in follow-up work. The main conclusions are as follows:

(1) Using the temperature, deformation, and residual stress data generated by the finite element simulation, multi-physics prediction models such as MLP were established. Under the current FEM-based and small-sample conditions, the MLP model performs well across all three physical fields: the average error in peak residual stress prediction is approximately 6 MPa, the test set Mean Absolute Error (MAE) for temperature and deformation prediction is the lowest, and the deviation for most samples is ≤5 MPa. The RF model ranks second, with an average peak residual stress prediction error of about 6.5 MPa and a sample deviation ≤ 8 MPa in most cases, showing satisfactory fitting performance.

(2) The results from 10-fold cross-validation show that the MLP model achieves the best overall performance and can effectively capture the nonlinear relationships between process parameters and multi-physics fields with the highest prediction accuracy (no significant overfitting and good generalization within the available dataset). However, limited by the small sample size, the model’s sensitivity under extreme welding conditions has not been fully verified, and its related performance needs to be further improved by expanding the dataset and conducting experimental tests in future work.

(3) The RF model (with performance only second to MLP) features a high training efficiency and strong interpretability, showing good potential for industrial applications. The RBF-SVR and TabNet models can meet basic prediction requirements but are not suitable for high-precision prediction tasks. Under the small-sample setting of this study, the XGBoost and FT-Transformer models yield relatively large prediction errors (peak residual stress error > 15 MPa, maximum deviation of extreme samples > 20 MPa). It should be clarified that these models are only unsuitable for the working conditions defined in this study, rather than being inapplicable to such application scenarios in general; their performance can be further verified under larger sample sizes or different task settings.

(4) The MLP model developed in this paper can effectively reproduce the variation laws of multi-physical fields revealed by finite element simulations and meet the basic engineering reference requirements. It can provide preliminary technical support for welding process monitoring and parameter optimization, while reducing the dependence on repeated FEM simulations.

Future work will expand the finite element simulation dataset by including more process and material conditions and supplementing independent experimental data for model calibration and validation. This will reduce simulation uncertainty, improve the model’s generalization ability and engineering reliability, and promote its practical application in industrial production.

## Figures and Tables

**Table 1:** Chemical composition of base material and filler material (wt.%) [29].

Material	C	Si	Mn	P	S	Ni	Cr	Cu	Mo	V	Fe
45Mn steel	0.46	0.24	0.71	0.016	0.006	-	0.0131	0.0097	-	-	Bal.
E5015	0.15	0.90	1.60	0.035	0.035	0.30	0.20	-	0.30	0.08	Bal.

**Table 2:** Range of welding process parameters.

Process Parameters	Symbol	Value
Welding current (A)	*I*	170–190
Arc voltage (V)	*U*	18–22
Welding speed (mm/s)	*v*	3–5
Torch incidence angle (°)	α	40–50
Welding mode	-	[1, 0], [0, 1]
Welding thermal efficiency	*η*	0.8

**Table 3:** Different combinations of welding process parameters.

Sample	Welding Current (A)	Arc Voltage (V)	Welding Speed (mm/s)	Torch Incidence Angle (°)	Welding Mode
1	185	21	4	43	[1, 0]
2	170	22	4	46	[1, 0]
…	…	…	…	…	…
50	170	21	3.5	43	[0, 1]
51	170	20	4.5	42	[0, 1]
…	…	…	…	…	…
99	175	22	4	50	[0, 1]
100	190	20	3	46	[0, 1]

**Table 4:** Core hyperparameters of machine learning models for temperature, deformation, and residual stress prediction in large flange shaft welding.

Model Type	Core Hyperparameters and Value
XGBoost	Booster = gbtree, learning_rate = 0.0001, n_estimators = 100, max_depth = 6, subsample = 0.8, colsample_bytree = 0.8, reg_alpha = 0.1, reg_lambda = 1.0, objective = reg:squarederror, random_state = 42, n_jobs = −1
RBF-SVR	Kernel function = Radial Basis Function (RBF), Penalty factor *C* = 10.0, gamma = scale, epsilon = 0.1, shrinking = True, random_state = 42, Kernel parameter *γ* = 0.01, Loss function *ε* = 0.1
RF	n_estimators = 100, max_depth = 8, min_samples_split = 2, min_samples_split = 2, min_samples_leaf = 1, max_features = sqrt, bootstrap = True, random_state = 42, n_jobs = −1

**Table 5:** MLP model hyperparameters for prediction of welding temperature, deformation, and residual stress in large flange shafts.

Hyperparameters	Value
Number of network layers	5
Number of neurons in the input layer	10
Activation function for the input layer	ReLU
Number of neurons in hidden layer 1	10
Activation function for hidden layer 1	ReLU
Number of neurons in hidden layer 2	8
Activation function for hidden layer 2	ReLU
Number of neurons in hidden layer 3	6
Activation function for hidden layer 3	ReLU
Number of neurons in the output layer	5
Activation function for the output layer	-
Loss function	MAE
Evaluation function	MAE MSE
Epochs	600
Batch size	3
Learning rate	0.0001

**Table 6:** Hyperparameters of deep learning models for welding temperature, deformation and residual stress prediction of large flange shafts.

Model Type	Core Hyperparameters and Value
TabNet	n_d = 8, n_a = 8, n_steps = 3, gamma = 1.3, n_independent = 2, n_share = 2, learning_rate = 0.02, batch_size = 8, max_epochs = 150, random_state = 42
FT-Transformer	d_model = 16, nhead = 2, num_layers = 1, dim_feedforward = 32, dropout = 0.1, learning_rate = 0.001, batch_size = 8, epochs = 150, loss = ‘MSE’, random_state = 42

**Table 7:** Process parameters for large flange shaft welding.

Variables	1	2	3	4
Welding current (A)	173	186	176	183
Arc voltage (V)	19.4	21.3	21.7	18.6
Welding speed (mm/s)	3.8	4.1	4.6	3.3
Torch incidence angle (°)	43	48	43	48
Welding mode	1	1	0	0

**Table 8:** Validation sample results of the MLP deep learning model.

Sample	1		2		3		4	
Feature	Actual	Predicted	Actual	Predicted	Actual	Predicted	Actual	Predicted
Weld Peak Temperature (°C)	2295.69	2205.00	2586.21	2512.96	3045.35	2926.53	3084.39	3000.14
Base Metal Peak Temperature (°C)	1394.68	1442.85	1576.53	1644.83	1617.77	1680.03	1797.06	1831.20
Deformation in X-direction (mm)	0.22	0.2	0.23	0.21	0.23	0.22	0.25	0.23
Deformation in Y-direction (mm)	0.27	0.24	0.24	0.26	0.23	0.25	0.28	0.27
Peak Residual Stress (MPa)	391.38	420.9	410.08	433.29	415.83	445.35	421.95	455.05

## Data Availability

The original contributions presented in this study are included in the article/Supplementary Material. Further inquiries can be directed to the corresponding author.

## Supplementary Materials

The following supporting information can be downloaded at: https://www.mdpi.com/article/10.3390/ma19050995/s1. Table S1: Different Combinations of Welding Process Parameters.

## Nomenclature

*I*	Welding current	$a_{f},a_{r}$	Lengths of the front and rear semi-axes in the double-ellipsoidal moving heat source model
*U*	Arc voltage	b,c	Parameter of the moving volumetric heat source
*v*	Welding speed	$\Delta \varepsilon_{\mathrm{total}}$	Total strain increment
*α*	Torch incidence angle	$\Delta \varepsilon_{\mathrm{elastic}}$	Elastic strain increment
*η*	Welding thermal efficiency	$\Delta \varepsilon_{\mathrm{plastic}}$	Plastic strain increment
ρ	Density	$\Delta \varepsilon_{\mathrm{thermal}}$	Thermal strain increment
$c_{p}$	Specific heat capacity	$\Delta \varepsilon_{\mathrm{creep}}$	Creep strain increment
k	Thermal conductivity	$\Delta \varepsilon_{\mathrm{phase}}$	Transformation strain increment
x,y,z	Coordinates	${\hat{y}}_{i}$	Predicted value
$Q\left(x,y,z,t\right)$	Internal heat generation rate	y	True value
T	Current temperature	n	Number of samples
t	Welding time	$l\left(y_{i},{\hat{y}}_{i}\right)$	Loss function for the *i*-th sample, and common loss functions
$h_{c}$	Convective heat transfer coefficient	$f_{k}$	*k*-th tree
ε	Radiant emissivity coefficient	$\Omega \left(f_{k}\right)$	Regularization term that controls the complexity of the tree
σ	Stefan–Boltzmann constant	*λ*	Regularization parameter
$T_{s}$	Ambient temperature	$w_{i}$	Weight of each leaf node in the tree
$q_{f}\left(x,y,z\right)$	Heat flux densities in the front part of the welding zone	ω	Weight vector
$q_{r}\left(x,y,z\right)$	Heat flux densities in the rear part of the welding zone	b	Bias term
Q	Heat source power	$\zeta ,\zeta^{*}$	Slack variables
$f_{f},f_{r}$	Front and rear energy distribution parameters	C	Penalty coefficient
$a^{l}$	Output of the *l*-th layer	$W^{l}$	Weight coefficient matrix of the *l*-th layer
$a^{l-1}$	Output of the (*l* − 1)-th layer and also the input of the *l*-th layer	$b^{l}$	Bias vector of the *l*-th layer
$z^{l}$	Linear computation result of the *l*-th layer		

## References

[1] Zhu Z., Wen C., Long T., Jin L., Li Y. (2023). Bifurcation analysis of a rotor-casing coupling system with bolted flange connection under the effect of rotor-casing rubbing fault. Processes.

[2] Xu Z., Yang C., Shi F., Liu W., Xu N., Hu Z., Li C., Liu K., Cao P., Wang D. (2025). Multiaxial Fatigue Life Assessment of Large Welded Flange Shafts: A Continuum Damage Mechanics Approach. Materials.

[3] Liu K.-l., Chen S.-c., Wang K., Yang D.-d., Chen M., Nie Z.-z., Zhang J. (2025). Research on the Application of High Torque Planar Friction Pair in Wind Turbine. Mech. Res. Appl..

[4] Zhou H., Lei K., Huang Z., Pang K., Qi F. (2025). Dynamic Simulation of Axle Box Bearing Fatigue in High-Speed Rail Systems Incorporating Train-Track Coupling Dynamics. Qual. Reliab. Eng. Int..

[5] Arora H., Kumar R., Gulati P., Sharma S., Dwivedi S.P., Saxena A., Kumar A., Sharma K., Kozak D., Hunjet A. (2024). Numerical simulation of temperature distribution and residual stress in TIG welding of stainless-steel single-pass flange butt joint using finite element analysis. High Temp. Mater. Process..

[6] Guan X., Hirohata M., Chang K.-H. (2025). A streamlined FE method for deformation and residual stress prediction in butt welding using continuous heat input. Weld. World.

[7] Visentin A., Campagnolo A., Babini V., Meneghetti G. (2024). Fatigue assessment of steel tube-to-flange welded joints with reinforcement ribs subjected to multiaxial loads according to the Peak Stress Method. Procedia Struct. Integr..

[8] Sun J., Nitschke-Pagel T., Dilger K. (2023). Generation and distribution mechanism of welding-induced residual stresses. J. Mater. Res. Technol..

[9] Falodun O., Oke S., Bodunrin M. (2025). A comprehensive review of residual stresses in carbon steel welding: Formation mechanisms, mitigation strategies, and advanced post-weld heat treatment techniques. Int. J. Adv. Manuf. Technol..

[10] Li Y., Li Y., Zhang C., Lei M., Luo J., Guo X., Deng D. (2022). Effect of structural restraint caused by the stiffener on welding residual stress and deformation in thick-plate T-joints. J. Mater. Res. Technol..

[11] Fu G., Lourenco M.I., Duan M., Estefen S.F. (2014). Effect of boundary conditions on residual stress and distortion in T-joint welds. J. Constr. Steel Res..

[12] Tang Y., Zheng L., Chen G., Xie D. (2026). Influence of plate thickness on welding induced distortion and residual stress of T-joints of DH36 marine steel. Ocean Eng..

[13] Kollár D. (2023). Numerical modelling on the influence of repair welding during manufacturing on residual stresses and distortions of T-joints. Results Eng..

[14] Khoshroyan A., Darvazi A.R. (2020). Effects of welding parameters and welding sequence on residual stress and distortion in Al6061-T6 aluminum alloy for T-shaped welded joint. Trans. Nonferrous Met. Soc. China.

[15] Ramos H., Tavares S., de Castro P. (2018). Numerical modelling of welded T-joint configurations using SYSWELD. Sci. Technol. Mater..

[16] Kumar B., Bag S. (2019). Phase transformation effect in distortion and residual stress of thin-sheet laser welded Ti-alloy. Opt. Lasers Eng..

[17] Kumar B., Bag S., Ruhul Amin M. (2022). Evaluation of phase Transformation strain and its influence on residual stress generation in laser welded Ti–6Al–4V alloy. J. Manuf. Sci. Eng..

[18] Cai W., Wang J., Zhou Q., Yang Y., Jiang P. (2019). Equipment and machine learning in welding monitoring: A short review. Proceedings of the 5th International Conference on Mechatronics and Robotics Engineering.

[19] Kumar S., Gaur V., Wu C. (2022). Machine learning for intelligent welding and manufacturing systems: Research progress and perspective review. Int. J. Adv. Manuf. Technol..

[20] Zhou B., Pychynski T., Reischl M., Kharlamov E., Mikut R. (2022). Machine learning with domain knowledge for predictive quality monitoring in resistance spot welding. J. Intell. Manuf..

[21] Song L., Zhang P., Chen K., Li Z., Yan H., Huang Y. (2025). From classical algorithms to deep learning: A review of machine vision for monitoring welding dynamics. Int. J. Adv. Manuf. Technol..

[22] Tessema S.H., Bismor D. (2024). Quality monitoring of hybrid welding processes: A comprehensive review. Arch. Control Sci..

[23] Lee J., Hwang H., Jeong T., Kim D., Ahn J., Lee G., Lee S.H. (2024). Review on Welding Process Monitoring Based on Deep Learning using Time-Series Data. J. Weld. Join..

[24] Yang T., Chen J., Chen S., Jiang Q., Li Y., Chen X., Tu Y., Xu Z. (2025). Prediction of residual stress field of ultrasonic rolling processed 20CrNiMo based on physics-informed neural networks. J. Manuf. Process..

[25] Xia Y.J., Song Q., Yi B., Lyu T., Sun Z., Li Y. (2025). Improving out-of-distribution generalization for online weld expulsion inspection using physics-informed neural networks. Weld. World.

[26] Qin Y., Ma C., Mei L. (2025). Prediction of weld residual stresses based on numerical simulation and machine learning: A review. Int. J. Adv. Manuf. Technol..

[27] Ng W.L., Goh G.L., Goh G.D., Sheuan J.T.J., Yeong W.Y. (2024). Progress and Opportunities for Machine Learning in Materials and Processes of Additive Manufacturing. Adv. Mater..

[28] Abderrachid H., Zohra B.F., Arvind A., Kang J., Hamid A. (2023). Advancements and applications of multiple wire processes in additive manufacturing: A comprehensive systematic review. Virtual Phys. Prototyp..

[29] Xu Z., Yang C., Liu W., Liu K., Shi F., Tan Z., Cao P., Wang D. (2025). Numerical Simulation of Arc Welding in Large Flange Shafts Based on a Novel Combined Heat Source Model. Materials.

[30] (2017). Hot-Rolled Quality Carbon Structural Steel Plates, Sheets and Strips.

[31] (2025). Specification for Carbon Steel Covered Electrodes for Shielded Metal Arc Welding.

[32] Hashemzadeh M., Chen B.Q., Soares C.G. (2015). Numerical and experimental study on butt weld with dissimilar thickness of thin stainless steel plate. Int. J. Adv. Manuf. Technol..

[33] Deng D., Kiyoshima S. (2011). FEM analysis of residual stress distribution near weld start/end location in thick plates. Comput. Mater. Sci..

[34] Sushil P., Pradeep R., Arvind K. (2021). A methodology to integrate melt pool convection with rapid solidification and undercooling kinetics in laser spot welding. Int. J. Heat Mass Transf..

[35] Mackerle J. (2003). Finite element analysis and simulation of polymers-an addendum: A bibliography (1996–2002). Model. Simul. Mater. Sci. Eng..

[36] Cheng L., Li Y., Wang J., Ma C., Zhan X. (2026). Prediction of laser welding deformation using a deep learning model optimized by a differential evolution algorithm. Chin. J. Mech. Eng..

[37] Chen T., Guestrin C. (2016). XGBoost: A Scalable Tree Boosting System. arXiv.

[38] Jiang B., Wang H., Zhou Y., Wang J. (2026). Deposition of coal dust particles in the human upper respiratory tract during unsteady breathing: Insights from CFD-DPM simulation and XGBoost-SHAP analysis. Powder Technol..

[39] Xing L., TianQiao L., Peng F. (2022). Long-term performance prediction framework based on XGBoost decision tree for pultruded FRP composites exposed to water, humidity and alkaline solution. Compos. Struct..

[40] Vapnik V.N. (2013). The Nature of Statistical Learning Theory.

[41] Lei L., Di L., Shuai R., HongGen Z., Jiasheng Z. (2021). Prediction of Welding Deformation and Residual Stress of a Thin Plate by Improved Support Vector Regression. Scanning.

[42] Luts J., Ojeda F., Plas R.V.d., Moor B.D., Huffel S.V., Suykens J.A.K. (2010). A tutorial on support vector machine-based methods for classification problems in chemometrics. Anal. Chim. Acta.

[43] Brand J., Zhang W., Carchman E., Dinh H.Q. (2026). Fluoro-forest: A random forest workflow for cell type annotation in high-dimensional immunofluorescence imaging with limited training data. Bioinform. Adv..

[44] Du B. (2026). Improving the accuracy of machine learning models in predicting evacuated tube solar collector through Random Forest replacement. Therm. Sci. Eng. Prog..

[45] Yasin K.H., Tulu D., Gelete T.B., Yuya B.A., Iguala A.D., Tadesse K.A., Kebede E. (2026). Random forest-based species distribution modeling reveals intensifying multi-species invasion risks of alien plants in Ethiopia under climate change. Remote Sens. Appl. Soc. Environ..

[46] Bergstra J., Bengio Y. (2012). Random Search for Hyper-Parameter Optimization. J. Mach. Learn. Res..

[47] Hidayaturrohman Q.A., Hanada E. (2025). A Comparative Analysis of Hyper-Parameter Optimization Methods for Predicting Heart Failure Outcomes. Appl. Sci..

[48] Balazs A., Miettinen J., Nilsson M., Breidenbach J., Pitkänen T.P., Myllymäki M. (2026). Large-scale forest resource mapping with spatial gaps in the training data: Comparison of different modeling approaches. Int. J. Appl. Earth Obs. Geoinf..

[49] Lim E., Lee H., Shin J.e., Yang H.J., Kim S.H., Kim S., Kim A. (2026). Multi-modal adaptive empathy assessment in online dyadic interaction using bi-directional multi-layer perceptron-mixer and dynamic weights fusion. Eng. Appl. Artif. Intell..

[50] Petrov S.M., Arapov D.V., Kuritsyn V.A., Podgornova N.M. (2026). Development of a high-accuracy multilayer perceptron-based soft sensor for real-time monitoring of supersaturation and dry substance content in vacuum pan crystallization. J. Food Eng..

[51] Jia Y., Wu J., Xu M. (2017). Traffic Flow Prediction with Rainfall Impact Using a Deep Learning Method. J. Adv. Transp..

[52] A N., V S. (2026). Autism spectrum disorder–level prediction and personalized education planning using TabNet. Autism.

[53] Termeh S.V.R., Niaraki A.S., Ali F., Pirasteh S., Choi S.M. (2025). Enhancing land subsidence susceptibility mapping using deep tabular learning optimization with metaheuristic algorithms. Gondwana Res..

[54] Zhao Z., Lin Q., Li Y., Li Y., Cui T. (2026). Stacking-based heterogeneous genetic programming for interpretable credit risk evaluation. Appl. Soft Comput..

[55] Haider J.Z., Ather A., ChangMin K., Jaegwan S., Jinwoo K., Changgil S., YongGu L., Sangwon K., Kangmin C., Hwa C.K. (2023). Transformer-based deep learning models for adsorption capacity prediction of heavy metal ions toward biochar-based adsorbents. J. Hazard. Mater..

[56] Wang X., Zhu Y., Wang P., Luo Y., Wei M., Wu Y. (2025). Research on FT-transformer-based intelligent discrimination model for roof stability classification of retreating coal roadways. Results Eng..

[57] Yan L. (2026). Energy consumption forecasting in logistics considering environmental and operational constraints using FT-transformer architecture. Sci. Rep..

